# Involvement of Akt and endothelial nitric oxide synthase in ventilation-induced neutrophil infiltration: a prospective, controlled animal experiment

**DOI:** 10.1186/cc6101

**Published:** 2007-08-23

**Authors:** Li-Fu Li, Shuen-Kuei Liao, Cheng-Huei Lee, Chung-Chi Huang, Deborah A Quinn

**Affiliations:** 1 Division of Pulmonary and Critical Care Medicine, Chang Gung Memorial Hospital, and Chang Gung University, Kweishan, Taoyuan 333, Taiwan; 2 Department of Respiratory Therapy, Chang Gung Memorial Hospital, Kweishan, Taoyuan 333, Taiwan; 3 Graduate Institute of Clinical Medical Sciences, Chang Gung University, Kweishan, Taoyuan 333, Taiwan; 4 Pulmonary and Critical Care Units, Department of Medicine, Massachusetts General Hospital, and Harvard Medical School, Massachusetts, USA; 5 Novartis Institute of Biomedical Research, Cambridge, Massachusetts, USA

## Abstract

**Introduction:**

Positive pressure ventilation with large tidal volumes has been shown to cause release of cytokines, including macrophage inflammatory protein-2 (MIP-2), a functional equivalent of human IL-8, and neutrophil infiltration. Hyperoxia has been shown to increase ventilator-induced lung injury, but the mechanisms regulating interaction between a large tidal volume and hyperoxia are unclear. We hypothesized that large tidal volume ventilation using hyperoxia would increase MIP-2 production and neutrophil infiltration via the serine/threonine kinase/protein kinase B (Akt) pathway and the endothelial nitric oxide synthase (eNOS) pathway.

**Methods:**

C57BL/6 mice were exposed to large tidal volume (30 ml/kg) mechanical ventilation with room air or hyperoxia for 1–5 hours.

**Results:**

Large tidal volume ventilation using hyperoxia induced neutrophil migration into the lung, MIP-2 production, and Akt and eNOS activation in a time-dependent manner. Both the large tidal volume ventilation of Akt mutant mice and the pharmacological inhibition of Akt with LY294002 attenuated neutrophil sequestration, MIP-2 protein production, and Akt and eNOS activation.

**Conclusion:**

We conclude that hyperoxia increased large tidal volume-induced MIP-2 production and neutrophil influx through activation of the Akt and eNOS pathways.

## Introduction

Acute respiratory distress syndrome (ARDS) is an inhomogeneous lung disease characterized by neutrophil influx into the lungs, by increased expression of inflammatory cytokines or chemokines, by loss of epithelial and endothelial integrity, and by the development of interstitial pulmonary edema [1]. The use of mechanical ventilation with high levels of oxygen to adequately oxygenate vital organs further increased the volutrauma and biotrauma of lungs supported by an increasing number of experimental and clinical data [2-6]. Mechanical ventilation with large tidal volume (V_T_) causes acute lung injury (ventilator-induced lung injury (VILI)) characterized by an inflammatory response morphologically and histologically indistinguishable from that caused by bacterial lipopolysaccharide [7,8]. Both large V_T _ventilation and hyperoxia alone can lead to the production of inflammatory cytokines including TNFα, IL-1β, and murine macrophage inflammatory protein-2 (MIP-2) [9-11], to airway apoptosis [12], to lung neutrophil influx [12], and to capillary leak [12]. IL-8 is a member of the cysteine–amino-cysteine chemokine family, and a potent chemoattractant for neutrophil recruitment associated with VILI in humans [13]. Murine MIP-2 is a functional homologue of human IL-8 in rodents and has been demonstrated to be a critical mediator in the pathogenesis of VILI [13]. The mechanisms of ventilator-induced inflammation and airway apoptosis with and without hyperoxia are complex, including activation of mitogen-activated protein kinases [12], serine/threonine kinase/protein kinase B (Akt), and endothelial nitric oxide synthase (eNOS) [14,15].

High V_T _ventilation can also lead to activation of Akt and eNOS [14,15]. Nitric oxide has been shown to be produced from L-arginine by a family of nitric oxide synthase isoforms, including inducible nitric oxide synthase and eNOS, which are expressed in a wide variety of tissues and cells [16]. Nitric oxide regulates smooth muscle cell relaxation, neurotransmission, macrophage-induced cytotoxicity, and induction of vascular and epithelial hyperpermeability [17,18]. The expression of eNOS may be induced by calcium-dependent binding of calmodulin via proinflammatory cytokines or by serine phosphorylation through the Akt pathway [19]. eNOS may mediate the systemic microvascular leak of VILI [20]. Phosphoinositide 3-OH kinase (PI3-K), a heterodimeric complex, and the downstream Akt have been shown to modulate neutrophil activation involved in acute lung injury [15].

In our previous work we have found that large V_T _ventilation results in increased lung neutrophil sequestration and increased MIP-2 production, which was, at least in part, dependent on the apoptosis signal-regulated kinase 1, c-Jun N-terminal kinase, and extracellular signal-regulated kinase 1/2 pathways [21]. In the present article we explore the hypothesis that large V_T _ventilation with hyperoxia induced MIP-2 production, and that neutrophil infiltration is dependent on the activation of the Akt and eNOS pathways.

## Materials and methods

### Experimental animals

Male C57BL/6 mice, either wild-type Akt^+/+ ^or Akt^+/- ^on a C57BL/6 background, weighing between 20 and 25 g were obtained from Jackson Laboratories (Bar Harbor, ME, USA) and the National Laboratory Animal Center (Taipei, Taiwan). Heterozygotes (+/-) are used because homozygotes exhibit lower fertility and female homozygotes do not nurse well; up to 50% perinatal mortality can occur. Mice that are heterozygous for the targeted mutation are viable and do not display any gross behavioral abnormalities.

The construct Akt containing disrupted exons 4–7 and the 5' end of exon 8 was electroporated into 129P2Ola/Hsd-derived E14 embryonic stem cells. Chimeras are generated by injecting these embryonic stem cells into C57BL/6 (B6) blastocysts. The resulting chimeric male animals were crossed to C57BL/6 mice, and then backcrossed to the same for 10 generations. Heterozygotes (+/-) are intercrossed to generate homozygous mutant mice (-/-) [22].

The lower expressions of the Akt protein in Akt^+/- ^mice were confirmed using western blot analysis. The study was performed in accordance with the animal experimental guidelines of the National Institutes of Health and with approval of the local research committee.

### Experimental groups

Animals were randomly distributed into seven groups in each experiment: group 1, control, nonventilated wild-type mice with room air (*n *= 6 each for western blot, Evans blue dye (EBD) assay, immunohistochemistry assay, and myeloperoxidase (MPO)/MIP-2); group 2, control, nonventilated wild-type mice with hyperoxia (*n *= 6 each for western blot, EBD assay, immunohistochemistry assay, and MPO/MIP-2); group 3, V_T _30 ml/kg wild-type mice with room air (*n *= 6 each for western blot: 60 min, 120 min and 300 min, eNOS inhibitor L-NAME (Sigma-Aldrich, St Louis, MO, USA), EBD assay, immunohistochemistry assay, and MPO/MIP-2); group 4, V_T _30 ml/kg wild-type mice with hyperoxia (*n *= 6 each for western blot: 60 min, 120 min and 300 min, L-NAME, EBD assay, immunohistochemistry assay, and MPO/MIP-2); group 5, V_T _30 ml/kg wild-type mice with LY294002 (*n *= 6); group 6, V_T _30 ml/kg Akt^+/- ^mice with room air (*n *= 6 each for western blot, EBD assay, immunohistochemistry assay, and MPO/MIP-2); and group 7, V_T _30 ml/kg Akt^+/- ^mice with hyperoxia (*n *= 6 each for western blot, EBD assay, immunohistochemistry assay, and MPO/MIP-2).

### Ventilator protocol

We used our established mouse model of VILI as previously described [21]. In brief, mice were ventilated with 30 ml/kg at 65 breaths/min for 1 and 5 hours while breathing room air or hyperoxia (>95% oxygen). Our previous work has shown that changes in cytokine production and neutrophil infiltration occur around 5 hours. Five hours of ventilation was therefore used for collection of samples of MIP-2, MPO, EBD leak, and immunohistochemical analyses [21]. At the end of the study period, heparinized blood was taken from the arterial line for analysis of arterial blood gas and the mice were sacrificed. After sacrifice, the lungs were lavaged and lung tissue was prepared for pathological examination or measurement of EBD extravasation, MPO activity, and kinase activation. Oxygen was fed into the inspiratory port of the ventilator when needed. Spontaneously breathing animals were exposed to hyperoxia in an enclosed chamber as previously described [2].

### Immunoblot analysis

Crude cell lysates were matched for protein concentration, resolved on a 10% bis-acrylamide gel, and electrotransferred to Immobilon-P membranes (Millipore Corp., Bedford, MA, USA). For assay of Akt and eNOS phosphorylation, western blot analyses were performed with antibodies to phospho-Akt and phospho-eNOS (New England BioLabs, Beverly, MA, USA). For determination of total Akt and eNOS protein expression, western blot analysis was performed with the respective antibodies (Santa Cruz Biotechnology, Santa Cruz, CA, USA). Blots were developed by enhanced chemiluminescence (NEN Life Science Products, Boston, MA, USA).

### Immunohistochemistry

The lung tissues from control, nonventilated mice, mice exposed to high V_T _ventilation for 5 hours while breathing room air or hyperoxia were paraffin embedded, sliced at 4 μm, deparaffinized, antigen unmasked in 10 mM sodium citrate (pH 6.0), and were incubated with phospho-Akt or phospho-eNOS primary antibody (1:100; New England BioLabs) and biotinylated goat anti-rabbit secondary antibody (1:100) according to the manufacturer's instruction of a immunohistochemical kit (Santa Cruz Biotechnology). The specimens were further conjugated with horseradish peroxidase–streptoavidin complex, detected by diaminobenzidine substrate mixture, and counterstained by hematoxylin. A dark-brown diaminobenzidine signal indicated positive staining of damaged epithelial cells, while shades of light blue signified nonreactive cells.

### Pharmacologic inhibitor

PI3-K inhibitor (LY294002; Sigma-Aldrich) 5 μg/g was given intraperitoneally 1 hour before ventilation, based on our dose–response studies that showed 5 μg/g inhibited Akt activity (data not shown). The eNOS inhibitor L-NAME (Sigma-Aldrich) 15 mg/kg was given intraperitoneally 1 hour before ventilation based on a previous *in vivo *study showing that 15 mg/kg inhibited eNOS activity [20].

### Statistical evaluation

The western blots were quantitated using a National Institutes of Health image analyzer (ImageJ 1.27z; National Institute of Health, Bethesda, MD, USA) and are presented as the ratio of phospho-Akt to Akt or of phospho-eNOS to eNOS (relative phosphorylation) in arbitrary units. Values are expressed as the mean ± standard error of the mean for at least three experiments. The data of MIP-2, MPO, EBD, and immunohistochemical analyses were analyzed using Statview 5.0 (Abascus Concepts Inc. and SAS Institute, Inc., Cary, NC, USA).

All results of western blot and MPO assays were normalized to control, nonventilated mice breathing room air. Analysis of variance was used to assess the statistical significance of the differences followed by multiple comparisons with a Scheffe' s test, and *P *< 0.05 was considered statistically significant.

EBD analysis, MPO assay, and measurements of MIP-2 were performed as previously described [12].

## Results

### Physiologic data

As we have shown previously [12], in the group of animals used for these experiments there was no statistical difference in pH, PaO_2_, PaCO_2_, mean arterial pressure, and peak inspiratory pressure found at the beginning versus at the end of 5 hours mechanical ventilation (Table 1). EBD analysis was used to measure changes of microvascular permeability in VILI. EBD was significantly higher in V_T _30 ml/kg mice with room air or hyperoxia compared with those of control, nonventilated mice (Table 1). EBD was attenuated in Akt mutant mice: V_T _30 ml/kg, wild-type, with room air, 76.8 ± 6.8 ng/mg versus V_T _30 ml/kg, Akt^+/-^, with room air, 43.9 ± 5.3 ng/mg (*P *< 0.05); and V_T _30 ml/kg, wild-type, with hyperoxia, 165.3 ± 8.4 ng/mg versus V_T _30 ml/kg, Akt^+/-^, with hyperoxia, 95.1 ± 6.0 ng/mg (*P *< 0.05).

**Table 1:** Physiologic conditions at the beginning and end of ventilation

	Nonventilated		Tidal volume 30 ml/kg	
	
	Room air	Hyperoxia	Room air	Hyperoxia
pH	7.40 ± 0.03	7.35 ± 0.01	7.33 ± 0.04	7.35 ± 0.03
PaO_2 _(mmHg)	98.7 ± 0.4	421.3 ± 5.3	86.1 ± 0.8	409.1 ± 4.1
PaCO_2 _(mmHg)	40.2 ± 0.3	39.1 ± 0.8	35.3 ± 1.4	43.1 ± 1.8
mean arterial pressure (mmHg)				
Start	86 ± 1.3	85.3 ± 2.1	84.6 ± 1.6	83.0 ± 2.8
End	85.2 ± 0.7	84.8 ± 0.9	73.5 ± 5.0	71.9 ± 4.3
Evans blue dye (ng/mg lung weight)	14.1 ± 1.3	15.9 ± 2.1	76.8 ± 4.7*	165.3 ± 7.9*

Arterial blood gases, mean arterial pressure, and Evans blue dye analysis of normal nonventilated mice and of mice placed on either room air or hyperoxia for 5 hours (*n *= 10/group). *P < 0.05 versus control, nonventilated mice.

### Lung stretch induced Akt and eNOS activation

**Figure 1:**
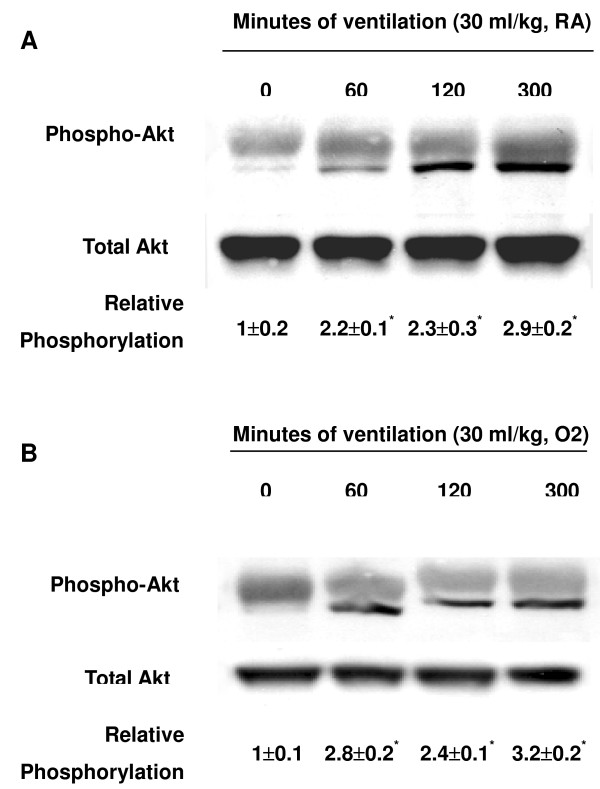
High tidal volume ventilation caused a time-dependent increase on Akt activation. Western blot was performed using an antibody that recognizes the phosphorylated serine/threonine kinase/protein kinase B (Akt) expression (**(a) **and **(b)**, top panel) and an antibody that recognizes total Akt protein expressions in lung tissue ((a) and (b), middle panel) from control nonventilated mice and from mice ventilated with tidal volume 30 ml/kg breathing room air or hyperoxia at indicated time periods. RA, mice with room air; O2, mice with hyperoxia. Arbitrary units are expressed as relative Akt phosphorylation ((a) and (b), bottom panel) (*n *= 6/group). *P < 0.05 versus control, nonventilated mice.

**Figure 2:**
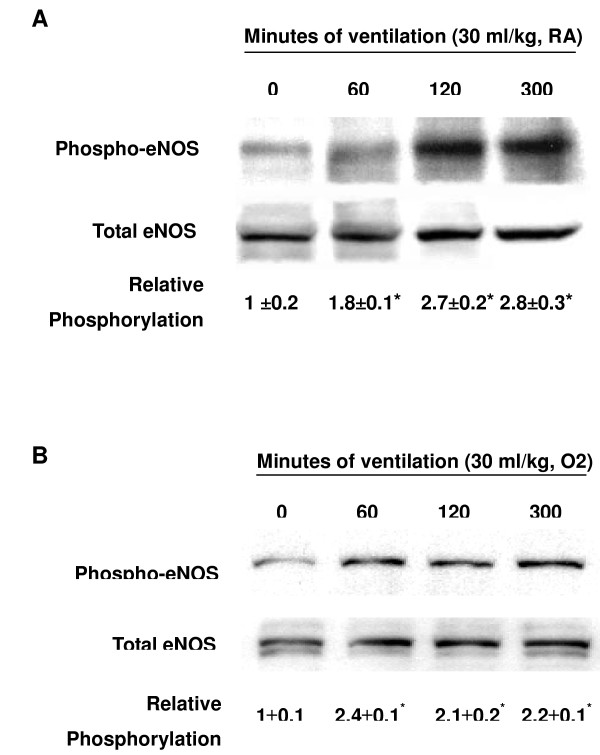
High tidal volume ventilation caused a time-dependent increase on endothelial nitric oxide synthase activation. Phosphorylated endothelial nitric oxide synthase (eNOS) expressions (**(a) **and **(b)**, top panel), total eNOS protein expressions ((a) and (b), middle panel), and relative phosphorylation quantitation by arbitrary units ((a) and (b), bottom panel) were obtained from control nonventilated mice and from mice ventilated with tidal volume 30 ml/kg using room air or hyperoxia at indicated time periods (*n *= 6/group). RA, mice with room air; O2, mice with hyperoxia. *P < 0.05 versus control, nonventilated mice.

**Figure 3:**
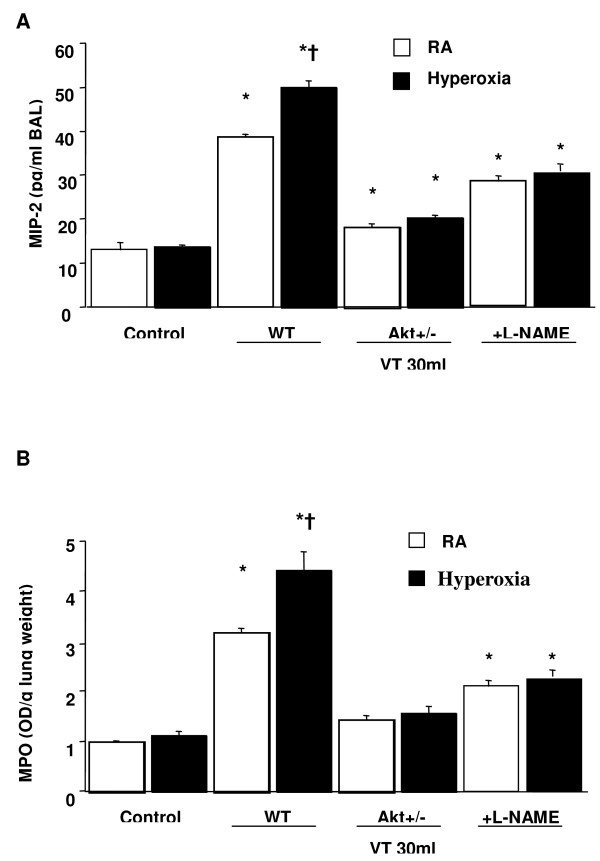
Effects of hyperoxia on stretch-induced infiltration of macrophage inflammatory protein-2 production and neutrophil influx. **(a) **Macrophage inflammatory protein-2 (MIP-2) production in bronchoalveolar lavage (BAL) fluid from control, nonventilated mice and from mice ventilated for 5 hours at tidal volume of 30 ml/kg with room air (RA) or hyperoxia (*n *= 6/group). **(b) **Myeloperoxidase (MPO) assay of lung tissue from control, nonventilated mice and from mice ventilated for 5 hours at tidal volume of 30 ml/kg with RA or hyperoxia (*n *= 6/group). L-NAME was given intraperitoneally (15 mg/kg) 1 hour before ventilation. *P < 0.05 versus control, nonventilated mice; †*P *< 0.05 versus all other groups. Akt, serine/threonine kinase/protein kinase B; OD, optical density; WT, wild-type.

We measured the activity of Akt and eNOS for 1–5 hours of mechanical ventilation to determine the time courses of stretch-induced kinase phosphorylation (Figures 1a and 2a). There were time-dependent increases in the phosphorylation of Akt and eNOS but there was no significant change in the expression of total nonphosphorylated proteins of Akt. Total nonphosphorylated eNOS increased, but less than that of phosphorylated eNOS. Both Akt and eNOS phosphorylation increased after 1 hour of mechanical ventilation with V_T _30 ml/kg and remained increased after 5 hours of mechanical ventilation as compared with control, nonventilated mice. This suggested that increases in the Akt and eNOS specific activity may be the mechanism of stretch-induced MIP-2 production and neutrophil infiltration (Figure 3).

### Inhibition of lung stretch-induced Akt and eNOS activation with LY294002

**Figure 4:**
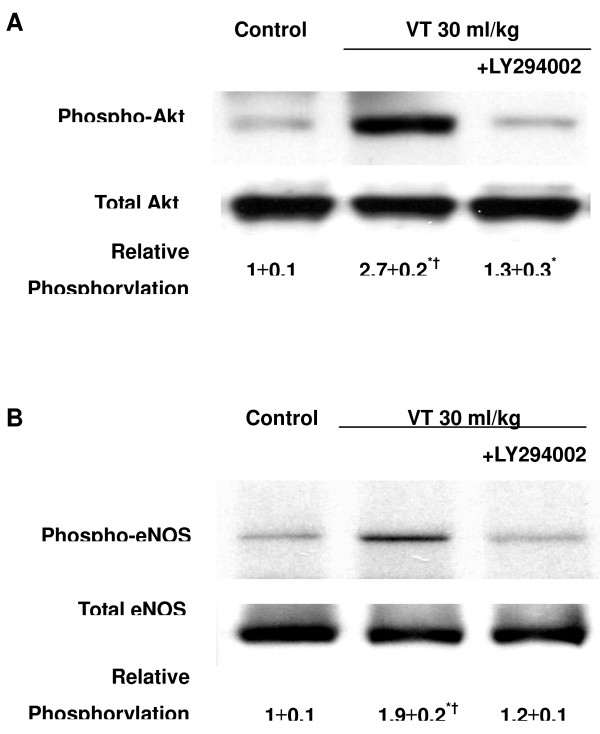
LY294002 reduced lung stretch-induced Akt and endothelial nitric oxide synthase activation. Mice ventilated at a tidal volume (V_T_) of 30 ml/kg for 1 hour were pretreated with 5 μg/g LY294002 intraperitoneally 1 hour before ventilation. Phosphorylated serine/threonine kinase/protein kinase B (Akt) or endothelial nitric oxide synthase (eNOS) expression (**(a) **and **(b)**, top panel), total Akt or eNOS protein expression ((a) and (b), middle panel), and relative phosphorylation quantitation by arbitrary units ((a) and (b), bottom panel) (*n *= 6/group). *P < 0.05 versus control, nonventilated mice; †*P *< 0.05 versus ventilation with LY294002.

To define the effectiveness of LY294002, a PI3-K inhibitor, on the stretch-induced Akt activation, we pretreated mice with LY294002 and performed western blot analyses to measure the phosphorylation of Akt and eNOS. LY294002 does not decrease total protein expression of Akt and eNOS but did significantly inhibit the large V_T _ventilation-induced activation of Akt and eNOS (Figure 4), suggesting that Akt and eNOS pathways may be involved in VILI.

### Effects of hyperoxia on lung stretch-induced Akt and eNOS activation

**Figure 5:**
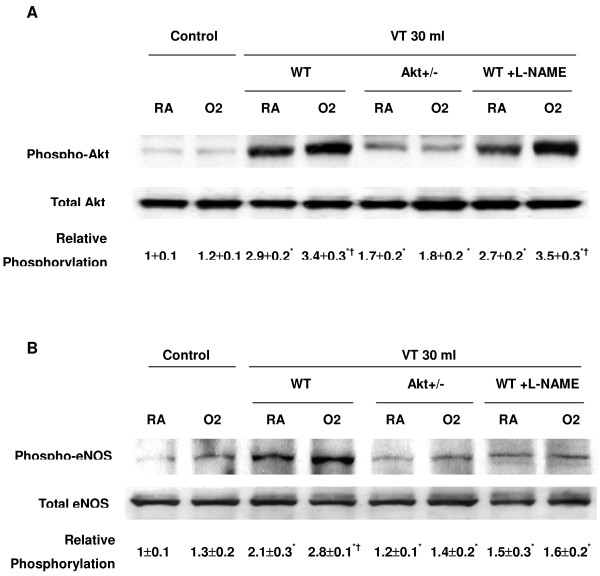
Akt mutants protected from hyperoxia effects on stretch-induced Akt and endothelial nitric oxide synthase activation. Phosphorylated serine/threonine kinase/protein kinase B (Akt) or endothelial nitric oxide synthase (eNOS) expressions (**(a) **and **(b)**, top panel), total Akt or eNOS protein expressions ((a) and (b), middle panel), and relative phosphorylation quantitation by arbitrary units ((a) and (b), bottom panel) were obtained from control nonventilated mice and from mice ventilated with tidal volume 30 ml/kg while breathing room air or hyperoxia for 1 hour (*n *= 6/group). L-NAME was given intraperitoneally (15 mg/kg) 1 hour before ventilation. WT, wild-type C57BL/6 mice; RA, mice with room air; O2, mice with hyperoxia. *P < 0.05 versus control, nonventilated mice; †*P *< 0.05 versus all other groups.

To determine the effects of hyperoxia on Akt and eNOS activation in VILI, we measured the activity of Akt and eNOS in mice exposed to V_T _30 ml/kg mechanical ventilation for 1–5 hours while breathing room air or hyperoxia (Figures 1b and 2b). Phosphorylation of both Akt and eNOS increased significantly after 1 hour of mechanical ventilation with V_T _30 ml/kg and remained sustained after 5 hours of mechanical ventilation as compared with control, nonventilated mice using hyperoxia. Mechanical ventilation with hyperoxia significantly augmented the activation of Akt and eNOS at 1 hour of ventilation as compared with mechanical ventilation with normoxia (Figure 5). No significant change was found in the expression of total nonphosphorylated proteins of Akt.

The targeted mutation gene of the Akt mutant is Akt1, and the Akt antibody used for the western blot assay reacted with Akt1, Akt2, and Akt3. The masking of specific Akt gene reduction by other isoforms explained the similar Akt expression levels in Akt^+/- ^mice and wild-type mice. The total nonphosphorylated eNOS increased but by less than that of phosphorylated eNOS. This suggests the addition of oxygen augmented the increases of the Akt and eNOS specific activity early (1 hour of ventilation) in the course of mechanical ventilation and may be involved in the mechanism of stretch-induced neutrophil infiltration (Figure 5). Mechanical ventilation for 1 hour was used in the rest of the experiments. The augmentation in eNOS activation is significantly less than that in Akt activation, suggesting the other pathway may be involved in the Akt activation using hyperoxia.

### Inhibition of Akt activation with Akt knockout mice reduced effects of hyperoxia on large tidal volume-induced eNOS activation

**Figure 6:**
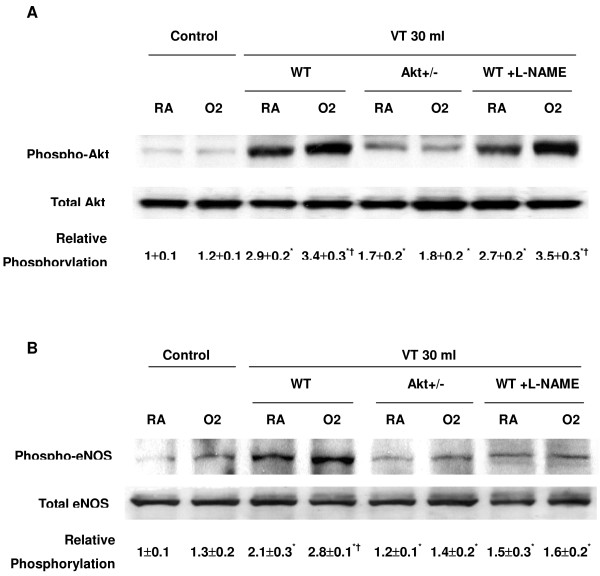
Effects of hyperoxia on stretch-induced Akt activation of airway epithelium in Akt mutant mice. Representative photomicrographs (×400) with phosphorylated serine/threonine kinase/protein kinase B (Akt) staining of the lung sections after 5 hours of mechanical ventilation with room air or hyperoxia (*n *= 6/group). **(a) **Control wild-type mice with room air. **(b) **Control wild-type mice with hyperoxia. **(c) **Tidal volume 30 ml/kg wild-type mice with room air. **(d) **Tidal volume 30 ml/kg wild-type mice with hyperoxia. **(e) **Tidal volume 30 ml/kg Akt^+/- ^mice with room air. **(f) **Tidal volume 30 ml/kg Akt^+/- ^mice with hyperoxia. A dark-brown diaminobenzidine signal indicates positive staining of lung epithelium, while lighter shades of bluish tan signify nonreactive cells.

**Figure 7:**
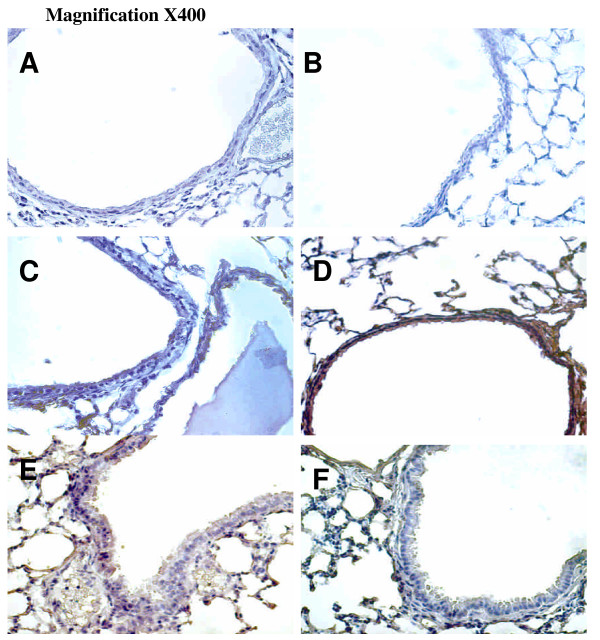
Effects of hyperoxia effects on stretch-induced endothelial nitric oxide synthase activation of airway epithelium. Representative photomicrographs (×400) with phosphorylated endothelial nitric oxide synthase staining of the lung sections after 5 hours of mechanical ventilation with room air or hyperoxia (*n *= 6/group). **(a) **Control wild-type mice with room air. **(b) **Control wild-type mice with hyperoxia. **(c) **Tidal volume 30 ml/kg wild-type mice with room air. **(d) **Tidal volume 30 ml/kg wild-type mice with hyperoxia. **(e) **Tidal volume 30 ml/kg Akt^+/- ^mice with room air. **(f) **Tidal volume 30 ml/kg Akt^+/- ^mice with hyperoxia. A dark-brown diaminobenzidine signal indicates positive staining of lung epithelium, while lighter shades of bluish tan signify nonreactive cells. Akt, serine/threonine kinase/protein kinase B.

To determine the role of Akt activation in ventilation-induced Akt and eNOS activation, we used Akt mutant mice. Heterozygous Akt mutant mice were ventilated at V_T _30 ml/kg for 1 hour. We confirmed the results of the western blot assay using immunohistochemistry, and identified the cell types in which large V_T _ventilation activated Akt and eNOS (Figures 6 and 7). Hyperoxia increased positive staining of phospho-Akt and phospho-eNOS in the airway epithelium of mice ventilated at V_T _30 ml/kg for 5 hours (Figures 6 and 7). The increases in positive staining of phospho-Akt and phospho-eNOS on the airway epithelium were reduced in Akt mutant mice. This added further evidence that hyperoxia-augmented lung stretch-induced lung inflammation was dependent, in part, on the Akt–eNOS pathway.

### Inhibition of Akt activation with Akt knockout mice reduced effects of hyperoxia on large tidal volume-induced infiltration of neutrophils and cytokine production

To determine the effects of hyperoxia on the upregulation of chemokines for neutrophils, and to determine the neutrophil content in the vasculature, in lung parenchyma, and in the alveoli, we measured MIP-2 protein production and MPO activity for 5 hours of mechanical ventilation (Figure 3). The MIP-2 and MPO levels in mice ventilated with hyperoxia at V_T _30 ml/kg were significantly elevated compared with control, nonventilated mice, and compared with mice ventilated with room air at V_T _30 ml/kg. Using Akt mutant mice receiving room air or hyperoxia at V_T _30 ml/kg mechanical ventilation, we found significantly decreased levels of MIP-2 and MPO in the Akt mutant mice. This observation suggested that addition of oxygen may be involved in large V_T_-induced neutrophil influx and MIP-2 production, and was dependent, in part, on the Akt–eNOS pathway.

## Discussion

Large V_T _in normal animals has been used to mimic the overdistention of the less injured and thus more compliant areas of the lung found in ARDS patients. These animal models, including our previous work, have shown that simply overdistending lung tissue, in the absence of any other stimuli, causes production of cytokines and chemokines, but the mechanisms have been unclear [1,8,21,23-25]. In a previous *in vivo *mouse study, we found that hyperoxia increased high V_T_-induced lung neutrophil sequestration and increased MIP-2 production, which was, at least in part, dependent on the c-Jun N-terminal kinase and extracellular signal-regulated kinase pathways [12]. We now show that activation of the Akt and eNOS pathways was also involved in ventilator-induced neutrophil infiltration and cytokine production with and without hyperoxia. With hyperoxia, however, the Akt and eNOS pathways were activated earlier in the course of high V_T _ventilation, and may have contributed to the increased lung injury seen in hyperoxia with high V_T _ventilation compared with high V_T _ventilation alone.

Large V_T _ventilation using hyperoxia has previously been shown in rat models to induce neutrophil migration into the alveoli and was dependent on MIP-2 production, a functional homologue of human IL-8 [2,11]. Hyperoxia alone had minimal effects on IL-8 production [9]. We found hyperoxia increased high V_T_-induced interstitial pulmonary edema of acute lung injury as measured by EBD (Table 1), neutrophil sequestration, and MIP-2 production (Figure 3). We explored further the pathways and cell types involved in this exacerbation of noncardiogenic pulmonary edema and lung inflammation.

The physical forces of mechanical ventilation are sensed and converted into the reactions of intracellular signal transduction via stress failure of the plasma membrane, stress failure of epithelial and endothelial barriers, mechanical stain, or shear stress [26]. Activation of PI3-K was demonstrated in endothelial cells by shear stress and in cardiac myocytes by stretch [27]. PI3-K and the downstream Akt play important roles in regulating neutrophil influx and chemotaxis [28,29]. Using mechanical ventilation, we found the addition of hypoxia augmented phosphorylation of Akt in a time-dependent manner (Figures 1 and 2). The contribution of Akt was further explored using a highly specific competitive inhibitor of PI3-K, LY294002, binding to the ATP-binding site (Figure 4) [30]. Using immunohistochemistry, we confirmed that large V_T _ventilation induced Akt activation in bronchial epithelial cells but not in endothelial cells and that Akt activation was augmented by adding hyperoxia (Figure 6). The discrepancies of cell types involved may be due to the different physical forces of mechanical strain and immunohistochemistry method limitations. Neutrophil sequestration to cysteine–amino-cysteine chemokines, such as IL-8, is dependent on PI3-K, apparently through mechanisms involving cytoskeletal reorganization [31].

Nitric oxide synthase can be induced in many cell types, including neutrophils and type II epithelial cells. eNOS has been shown to be a target of Akt, and inhibition of the PI3-K and Akt pathway or mutation of the Akt site on eNOS protein (at serine 1,177) attenuated the serine phosphorylation and prevented the activation of eNOS [19]. We found large V_T _ventilation increased eNOS phosphorylation in bronchial epithelial cells, neutrophil infiltration, and MIP-2 protein production (Figures 1, 2, and 7). These effects were augmented after adding hyperoxia but were blocked in Akt mutant mice (Figures 3 and 5).

**Figure 8:**
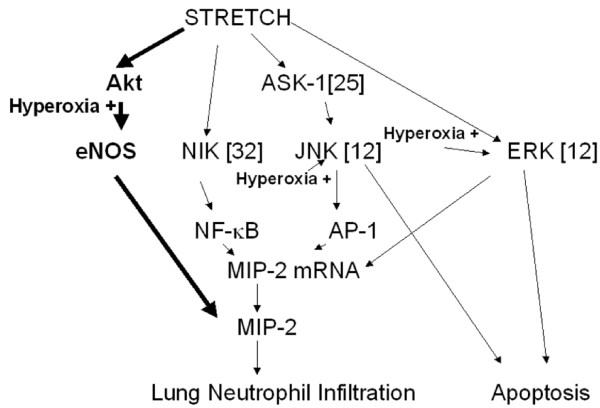
Differences in signaling pathway activation of mechanical ventilation with and without hyperoxia. In previous *in vitro *and *in vivo *studies we found ventilation-induced activation of apoptosis signal-regulated kinase 1 (ASK1), nuclear factor-κB-inducing kinase (NIK), c-Jun N-terminal kinase (JNK) and extracellular signal-regulated kinase (ERK) pathways [12,25,32]. In the present study, we found that activation of the serine/threonine kinase/protein kinase B (Akt) and endothelial nitric oxide synthase (eNOS) pathways was also involved in ventilator-induced neutrophil infiltration, cytokine production, and microvascular permeability with and without hyperoxia. MIP-2 = macrophage inflammatory protein-2; NF = nuclear factor.

Findings in other studies support our findings that neutrophil infiltration and the development of acute lung injury involve the PI3-K and Akt pathway in an isolated mouse model of endotoxemia [14,15]. Dimmeler and colleagues exposed human umbilical vein endothelial cells to shear stress in a cone-plate viscometer [19], and found activation of eNOS in endothelial cells by Akt-dependent phosphorylation via a Ca^2+^-independent mechanism. Other workers in our research group have found that eNOS but not inducible nitric oxide synthase may mediate the systemic microvascular leak in a rat model of VILI [20]. We found mechanical ventilation to cause phosphorylation of eNOS and the upstream regulator of Akt with or without hyperoxia; however, hyperoxia augmented activation of Akt/eNOS early in the course of ventilation (Figure 8).

In the clinical daily practice of ARDS, patients receive a longer duration of hyperoxia than in this experiment; further experiments using an *ex vivo *or *in vitro *model may therefore explore more about the effects of hyperoxia. Furthermore, significantly less augmentation of eNOS than that in Akt and the discrepancy of cell types involved in our study suggested the use of a single model may be limiting in terms of providing adequate generalizable information.

## Conclusion

Using an *in vivo *mouse model, we have found that hyperoxia increased high V_T_-induced epithelial cell injury, resulted in increased pulmonary neutrophil sequestration, and increased MIP-2 production, which was, at least in part, dependent, on the Akt and eNOS pathways. In subjects with severe ARDS the V_T _cannot be lowered to the recommended 6 ml/kg, and hyperoxia is required to maintain oxygenation. These data have added to the understanding of the mechanism involved in the effects of mechanical forces in the lung with hyperoxia, and have advanced the growing knowledge of the biochemical pathways involved in the pathogenesis of biotrauma with hyperoxia.

## Key messages

• Hyperoxia augments VILI.

• Hyperoxia augmentation of VILI depends on Akt and eNOS activation.

• Inhibition of Akt and eNOS may offer new treatment options for patients with severe ARDS.

## Abbreviations

Akt = serine/threonine kinase/protein kinase B; ARDS = acute respiratory distress syndrome; EBD = Evans blue dye; eNOS = endothelial nitric oxide synthase; IL = interleukin; MIP-2 = macrophage inflammatory protein-2; MPO = myeloperoxidase; PaCO_2 _= arterial carbon dioxide pressure; PaO_2 _= arterial oxygen pressure; PI3-K = phosphoinositide 3-OH kinase; TNF = tumor necrosis factor; VILI = ventilator-induced lung injury; V_T _= tidal volume.

## Competing interests

DAQ is an Assistant Professor of Medicine at Harvard Medical School, an Associated Physician at Massachusetts General Hospital, and an employee of Novartis Pharmaceuticals. Novartis Pharmaceuticals was otherwise not involved in this research and did not contribute to the funding for this project. All other authors declare that they have no competing interests.

## Authors' contributions

L-FL collected and analyzed the data. DAQ, S-KL, C-CH and C-HL reviewed and coordinated the study.
